# Initial Results of the Master's Degree Programme in "Leadership in Medicine" – Impact on hospital-based follow-on training of doctors

**DOI:** 10.3205/zma001129

**Published:** 2017-11-15

**Authors:** Chris-Henrik Wulfert, Joachim Hoitz, Ulrike Senger

**Affiliations:** 1 Federal Armed Forces Hospital Hamburg, Department of General, Visceral and Thoracic Surgery, Hamburg, Germany; 2 Federal Armed Forces Hospital Hamburg, Hamburg, Germany; 3 Helmut Schmidt University/Bundeswehr University, Centre for Postgraduate Education, Hamburg, Germany; 4 TU Dortmund University, Higher Education, Dortmund, Germany

**Keywords:** Postgraduate medical education, competency-based education, interpersonal and communication skills, professional competency, leadership, academic training, curricula, teaching methods

## Abstract

**Objective: **This pilot project, which was jointly conducted by a hospital and a university, describes the development of the Master's Degree Programme in Leadership in Medicine, a course designed to supplement medical specialty training. The aim of the pilot project is to demonstrate how hospital-based projects on personnel and organisational development undertaken under academic supervision can be used to increase leadership responsibility among doctors whose duties include providing initial and follow-on training and to professionalise medical specialty training as a leadership task. This need arose from the nationwide requirements and an internal audit regarding follow-on training. The version of the degree programme described below aims to further the personnel development of the participants in the field of didactics.

**Method: **Each of the nine modules is made up of two classroom-based phases and one distance learning phase. The distance learning phase involves undertaking hospital-based projects on personnel and organisational development under academic supervision. The pilot phase participants were hospital doctors who, as part of their duties, hold leadership responsibility or are involved in the follow-on training of doctors.

**Results:** The 17 participants successfully implemented more than 30 hospital-based projects during the distance learning phases of the nine modules. These projects included the development of medical specialty curricula, relevant didactic methods and evaluation design and were subsequently presented and subjected to reflection in interdisciplinary groups. The project presentation together with the project report were regarded as proof of competency.

**Conclusion:** In addition to enhancing participant competency, the degree model described, which interlinks theory and practice, promotes organisational development through the implementation of projects undertaken under academic supervision. This has a double impact on the quality of medical follow-on training at the hospital where the participant is based, for not only is the individual's didactic competency enhanced, but so is the "learning organisation" as a whole as a result of continuous project orientation.

## Zusammenfassung

**Zielsetzung: **Das vorliegende Pilotprojekt eines Krankenhauses und einer Hochschule beschreibt die Entwicklung eines die Facharztweiterbildung ergänzenden Weiterbildungsmasters „Führung in der Medizin“ (M-FIM). Es soll zeigen, wie durch wissenschaftlich begleitete Projekte zur Personal- und Organisationsentwicklung einer medizinischen Einrichtung die Führungsverantwortung von Aus- und Weiterbildern gestärkt und die Facharztweiterbildung als Führungsaufgabe professionalisiert werden kann. Die Notwendigkeit dazu ergab sich aus den bundesweiten Vorgaben und der hauseigenen Evaluation zur Weiterbildung. In der hier beschriebenen Studiengangsvariante wird eine didaktische Personalentwicklung der Teilnehmenden angestrebt.

**Methodik: **Jedes der 9 Studienmodule besteht aus zwei Präsenz- und einer Fernlernphase. In dieser werden Projekte zur Personal- und Organisationsentwicklung innerhalb des Krankenhauses erarbeitet und wissenschaftlich begleitet. Die Teilnehmer der Pilotphase rekrutierten sich aus Ärzten des Krankenhauses, die im Rahmen ihrer Tätigkeit Führungsverantwortung oder Aufgaben in der ärztlichen Weiterbildung wahrnehmen.

**Ergebnisse: **Von den 17 Teilnehmern konnten während der Fernlernphasen der 9 Module mehr als 30 Projekte im Krankenhaus umgesetzt werden. Es wurden unter anderem Facharztcurricula, entsprechende didaktische Methoden und Evaluationsdesigns entwickelt, in der interdisziplinären Gruppe präsentiert und reflektiert. Als Kompetenznachweis dienen dabei die Projektpräsentation und ein Projektbericht.

**Schlussfolgerung: **Das dargestellte Studienmodell der Theorie-Praxis-Verzahnung fördert neben dem Kompetenzzuwachs der Teilnehmenden über das wissenschaftlich begleitete Arbeiten die Organisationsentwicklung durch die erarbeiteten Projekte. Dies hat doppelten Einfluss auf die Qualität der ärztlichen Weiterbildung der entsendenden Einrichtung. Denn es wird nicht nur die didaktische Kompetenz des einzelnen gestärkt, sondern durch den kontinuierlichen Projektbezug die „lernende Organisation“ insgesamt.

## 1. Introduction

The issue of medical specialist competency in the modern healthcare system, and how it can be achieved, has already been raised in this journal and elsewhere on numerous occasions in recent years [1], [2], [3]. In fact, the reform of the (Model) Specialty Training Regulations towards a focus on competency, which has been ongoing since 2010, is now at an advanced stage [4]. These efforts were prompted by calls to make initial and follow-on training of doctors comparable at the European level by establishing educational standards [5] and implementing quality criteria with verifiable outcomes [1], [6], [7]. 

The implementation of the National Competency-Based Catalogue of Learning Objectives for Medicine by the German Association of Medical Faculties was undoubtedly a decisive step in the continuous process of lifelong learning that is part of every doctor's professional development [8]. In this catalogue of objectives, it was agreed that the outcome of medical training should conform to the role concept of the CanMEDS framework [9]. Our neighbouring countries, Switzerland and the Netherlands, have also joined this framework [10], [11]. The authors take the view that it is only logical that these efforts should also extend to medical specialty training.

The question that now arises, however, is how these aims can be developed during medical specialty training and subsequently achieved. The CanMEDS role of “medical expert” is adequately represented in the current Specialty Training Regulations, at least in terms of content. The specialty knowledge contents and technical abilities listed in appropriate detail. Imparting and evaluating these capabilities, however, is an increasing challenge for hospital-based medical specialists responsible for providing follow-on training [12]. This is where the other CanMEDS framework roles factor into the framework [13]. Today doctors, including those far below senior consultant level, spend a considerable proportion of their working hours on organisational and leadership tasks [9], [14]. These include supporting follow-on and advanced training, management competency in hospitals and community-based medical practices, communication, moderating, motivating, personnel and team leadership tasks as well as setting an example with regard to attitude and social skills [3]. Although the leadership role of doctors is considered to be of increasing significance, it is still only of secondary importance in most curricula governing the initial and follow-on training of doctors. The pertinent training concepts evaluated are limited to imparting theoretical knowledge and lack practical relevance [15]. In the revised version of the CanMEDS Framework 2015, the role of “manager” was replaced by that of “leader”, thus attaching appropriate importance to the competencies described therein [16]. 

Hence, this pilot project “Leadership in Medicine” aims both to bring about a verifiable increase in competencies among participants in their leadership role as a medical specialist responsible for providing initial and follow-on training and, at the same time, to ensure the professionalisation of medical specialty follow-on training through academic reflection on everyday clinical practice. This is to be achieved through work within the different modules that is project-based and scientifically supported and, in addition to an individual project report, will produce results that have been evaluated in practice.

## 2. Project Description

### 2.1. Development Milestones

The Federal Armed Forces Hospital Hamburg regards itself as a training institution committed to the Medical Service's mission of training and maintaining the proficiency of medical personnel, thus enabling them to provide medical care in Germany and, in particular, on operations abroad. 

On the basis of the German Medical Association's efforts to evaluate the follow-on training of doctors in 2009 and 2011 [17], the Department of Internal Medicine of the Federal Armed Forces Hospital Hamburg introduced a mentoring concept for junior doctors joining the hospital after graduation. The aim is to improve evaluation outcomes through structured introductory training and support. The areas of “imparting specialist competency”, “learning culture”, “leadership culture” and “decision-making culture” were identified as requiring improvement. The project was supervised by the Professor of General Education at Helmut Schmidt University/Bundeswehr University, Hamburg as part of a Master's dissertation [18]. 

Following on from the assessment carried out by the medical associations, in which only certain departments of the Federal Armed Forces Hospital Hamburg participated, a survey on the quality of medical follow-on training was conducted in all departments of the hospital that provide curative care. As with the national outcomes, a very mixed picture emerged and potential for development within the hospital became apparent. The structural framework was regarded as more or less in need of improvement. This was particularly evident from the statements “The structures in my department are definitely geared towards the training of doctors” and “There is a good balance between normal work and training” being rated an average of 3.17 on the German academic grading scale of 1 (very good) to 6 (inadequate). As with the national study, the ratings awarded for “academic basics” of medical specialty training were far lower than for other areas, ranging between 3 and 4 [19]. These outcomes resulted in two sets of measures being developed. Firstly, doctors specially authorised to provide follow-on training and doctors assigned follow-on training tasks in the individual departments (usually medical specialists and consultants, but also junior doctors at an advanced stage of their medical specialty training) were to receive didactic professionalisation training in a “train the trainer” approach. Secondly, medical specialty training, as a personnel development tool, was to be made subject to quality management. For this reason, the Centre for Postgraduate Education of the Helmut Schmidt University/Bundeswehr University, Hamburg was brought on board as a partner to develop and implement the Master's Degree Programme in Leadership in Medicine, a degree programme closely reflecting everyday clinical practice. In keeping with the strategic focus, the participants underwent personnel development measures in the area of medical initial and follow-on training. Based on these aims, the Centre for Postgraduate Education developed project-based follow-on training modules, which were then implemented in close interinstitutional cooperation with the Federal Armed Forces Hospital Hamburg. This ultimately led to the launch of a now successfully accredited modular Master's degree programme that carries 60 or 90 ECTS points [20], [21], [22].

#### 2.2. Objective of the Degree Programme

The objective of the curriculum for the Master's Degree Programme in Leadership in Medicine is the holistic and interdisciplinary training of leaders in medical professions and to facilitate an academic approach by incorporating educational sciences, social sciences, economics, law and even engineering. The subject-specific and interdisciplinary methods applied to academic work set the quality standard just as much as the continuous interlinking of theory and practice in everyday clinical practice, which, in terms of design and implementation, is particularly put to the test and evaluated when applied in a practical setting [20].

#### 2.3. Structure/Modular Structure

**Figure 1:**
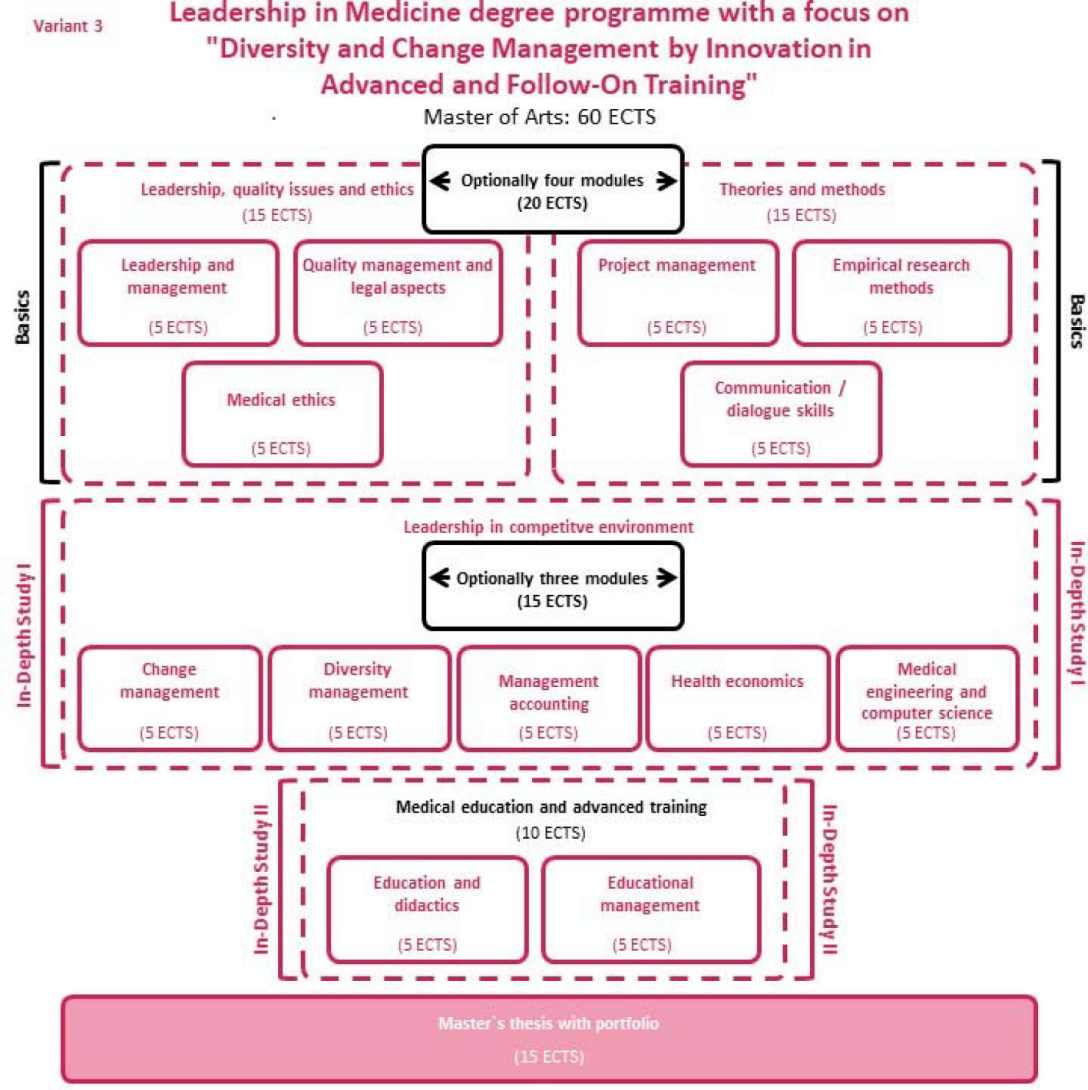
Degree programme model "Diversity and Change Management by Innovation in Advanced and Follow-On Training"

In addition to the general curriculum, three versions of the degree programme are offered. These consist of “Leadership and Management”, “Leadership with an Emphasis on Personnel Management/Advancement of Junior Personnel” and the current pilot version, “Diversity and Change Management by Innovation in Follow-On and Advanced Training” (see Figure 1 (Fig. 1)). 

The fundamental part of the course allows participants to acquire academic knowledge regarding leadership, quality management and law and, directly related to this, the ability to grasp and acquire academic theories and methods. The design, execution and evaluation of models of quantitative and qualitative empirical social research processes are of key importance. In line with the institution's requirements regarding follow-on training and change, the participants independently (under academic supervision) identify and prepare appropriate research designs for integrating quantitative and qualitative methods, especially with the aim of surveying perceptions from a multitude of perspectives to thus develop specific action and leadership measures and ensure their implementation in effective project management. Acquiring participative communication strategies is just as important as the ability to perform legally sound quality management [20].

#### 2.4. Cycle of Institution-Based Innovation and Quality Management

**Figure 2:**
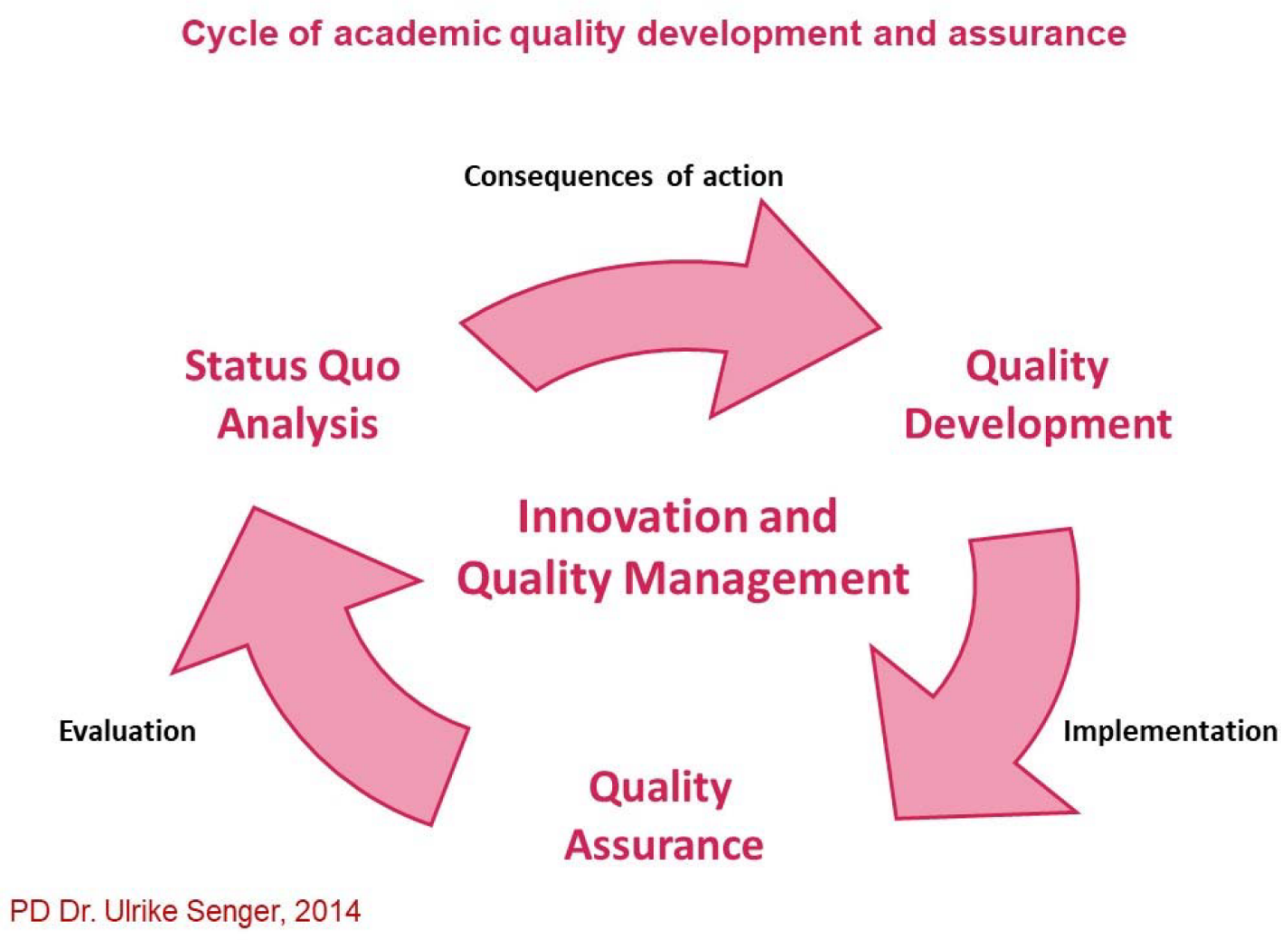
Cycle of academic quality development and assurance according to Senger [23]

The quality criterion for the acceptance and sustainable consolidation of innovative “project processes and products” is their successful integration in existing structures. To this end, the Master's Degree Programme in Leadership in Medicine makes use of the cycle of institution-based innovation and quality management (see Figure 2 (Fig. 2)) which, on the basis of a criteria-led analysis of the status quo, identifies possible development potential and action consequences and develops and tests needs-oriented pilot processes and projects for the purpose of quality development. The implementation phase is conceived as quality assurance in professional practice, with professional practice undergoing continuous evaluation on the basis of the quality criteria used in the status quo analysis and in project development [23]. 

Throughout a module, the participants work their way through this cycle and thus provide a basis for the continuous future development of the organisation by future generations.

#### 2.5. Module Description Based on "Educational Management" as an Example

The CanMEDS role of “scholar” and particularly the subdomain of medical didactics are not given much weight in the current Specialty Training Regulations. Moreover, the regulations on follow-on training do not define clearly enough what is required of doctors authorised to provide such training, and the framework conditions are far too outdated to merit the term “professionalization” [1], [12], [24], [25]. 

The module design described below outlines the didactic aims and contextual conditions that are of key importance for the professionalisation concept. 

##### 2.5.1. Competency Objectives as Defined in the Module Handbook 

The participants are familiar with education, knowledge and competency development, both in terms of concept and content. This allows them to reflect on their roles and attitudes as learners and teachers. They develop and evaluate competency-oriented formats for learning, teaching, counselling and assessment, which they implement in “constructive alignment”, taking medical specialty training as an example [26]. In this context, the participants test and evaluate the use of different didactic methods and formats, such as problem-based learning, enquiry-based learning, self-reflection and self-evaluation or mentoring. 

The outcomes enable them to develop a didactic professionalisation concept for doctors responsible for providing follow-on training and to create an appropriate pool of didactic methods to which they add recommendations and innovative practical examples taken from medical training practice. They base their didactic thinking and action on an institution-specific charter on teaching quality development and assurance [20].

##### 2.5.2. Implementation

Each module carries 5 ECTS credits, which corresponds to a commitment of 125 teaching units of 45 minutes each.

The first classroom-based phase comprises 20 teaching units over a period of three days (Thursday to Saturday) and serves as an introduction to the conceptual study of education, knowledge, learning and competency development as well as educational and learning theories. One focus is on the study of competency development and competency structure models. The European Qualifications Framework [27] and the German Qualifications Framework [28] for Lifelong Learning are taken into account and participants are provided with a repertoire of didactic methods of competency-oriented teaching, counselling and assessment. The participants evaluate the effectiveness and sustainability of these methods in medical specialty training on the basis of relevant criteria. When dealing with the topic of learning and curricular development, the concept of “constructive alignment” is introduced and applied to different case scenarios [26]. 

The participants use this as a basis for developing a professionalisation concept for doctors authorised to provide training or doctors assigned training tasks, meeting in project groups to decide on the objectives of and a plan for the forthcoming distance learning phase. During this distance learning phase, the course participants develop both a competency-oriented curriculum and a pool of didactic methods that includes case studies taken from clinical practice. The participants structure this pool of methods and compile it to serve trainees and trainers in medical specialty training. The pool is to be further developed after completion of the module and provide a platform for exchanging information on modern follow-on training in various medical specialties. 

The second classroom-based phase also comprises 20 teaching units. It focuses on the further development of the overall concept of the pool of didactic methods and the process of adding and further delving into case studies drawn from teaching and mentoring. The projects developed by the individual groups are presented to and evaluated by the entire peer group. In this setting, incentives and acceptance issues associated with both the handling and successive expansion of the set of instruments are reflected on and discussed [20].

The module ends with the assessment of the “Entrustable Professional Activities” workshop, which provided participants with an opportunity for exchange of information with two representatives of the German Association for Medical Education and external feedback on their current efforts [7].

## 3. Module/Project Completion

Nine modules have been completed since autumn 2013, with more than 30 projects implemented, many addressing the organisational framework or specifically initial and follow-on training issues (see Table 1 (Tab. 1)). The Master's theses based thereon are currently under review.

### 3.1. Participants

A total of 17 participants registered for the pilot project, 14 of whom completed all nine modules in full and submitted their Master's theses. Two participants joined the degree programme at a later date. The participants included specialists in internal medicine, neurology, oral and maxillofacial surgery, urology, general and visceral surgery, anaesthesiology, otolaryngology and dentistry as well as one pharmacist and the hospital's financial controller. There were two senior consultants, eight consultants and two junior doctors at an advanced stage of medical specialty training. One participant left the degree programme to pursue new career interests elsewhere. 

#### 3.2. Implemented Projects

At least three projects or seminar papers were completed in each of the modules so far, either in small groups or by individual participants. They all focused on the requirements identified by the participants in the status quo analysis of their organisation (see Table 1 (Tab. 1)). A group's presentation of the project outcomes and the preparation of a project report or seminar paper, which is added to the transcripts of records that serves as a logbook, are considered proof of competency. Assessment did not take the form of written examinations.

**Figure 3:**
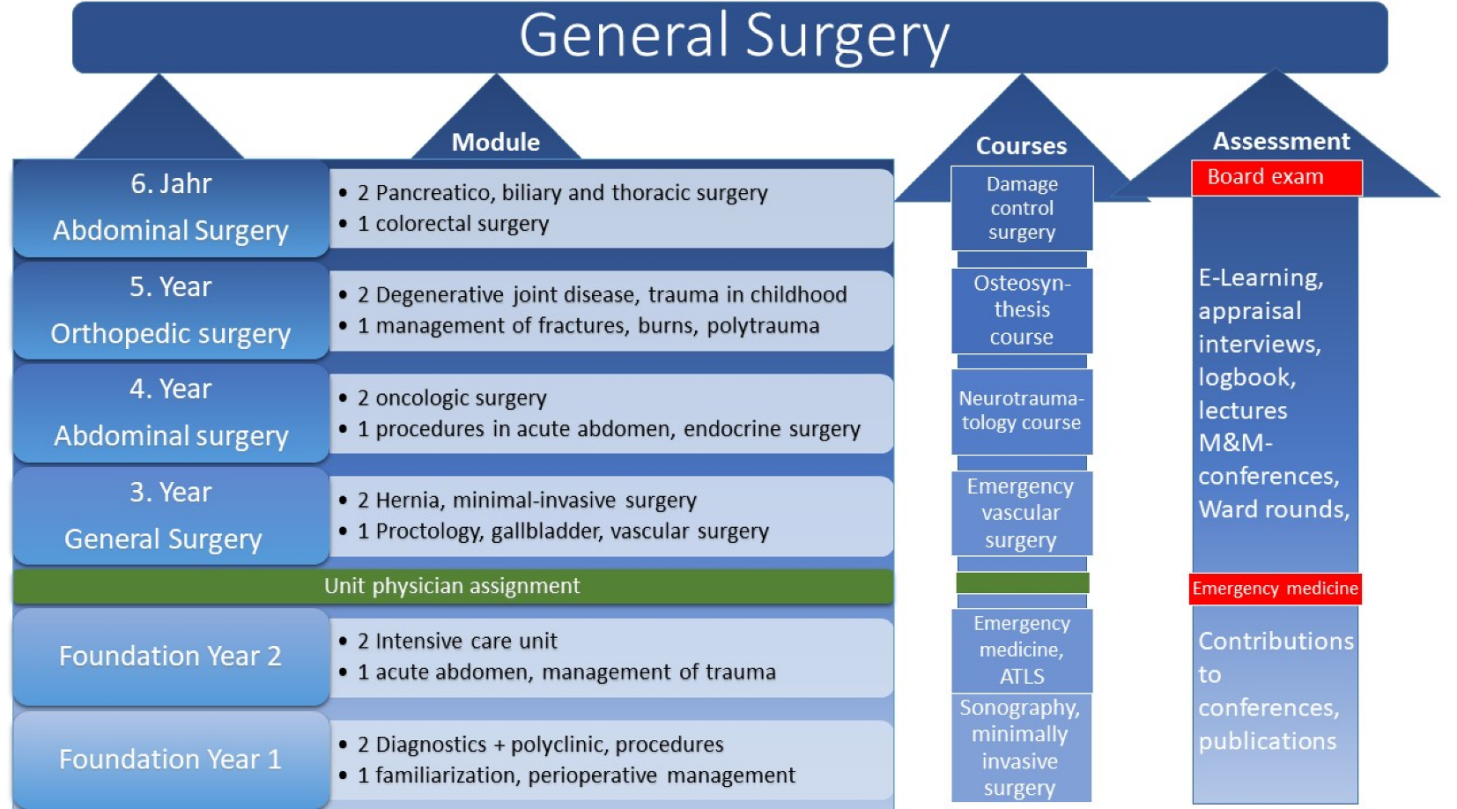
Project Product "Curriculum for Medical Specialty Training in General Surgery"

The pilot run saw the development of competency-based follow-on training concepts of differing characteristics for the various departments, for which the participants drew on current medical-didactic developments, such as the CanMEDS Framework [29]. Figure 3 (Fig. 3) shows an example of a “project product” as developed by a study group. It shows a structure developed for medical specialty training in general surgery that includes topics, assigned courses/workshops as well as evaluation options. The curriculum and its learning objectives were uploaded to the ILIAS electronic learning platform of the Bundeswehr University, Hamburg, and linked to reading recommendations and a list of audiovisual media. A peculiarity of surgical training in the Bundeswehr, besides military-surgical aspects, is that it is interrupted by a stint as a unit physician, during which no surgical training is provided and which, given that it takes place outside the hospital setting, is more centred on general medicine. 

In addition to the empirical project design, the participants develop criteria to evaluate their project papers as objectively as possible. These criteria serve as the basis for peer feedback as well as the institutional assessment of quality and sustainability. In the case study described, regular status quo analyses or competency checks in line with case studies from everyday clinical practice are to be conducted on a regular basis. Acceptance is also a criterion and can be measured by the number of participants and their qualitative and quantitative performance. 

The change process, which is defined and, with regard to its feasibility and effectiveness, reflected by milestones, must always be taken into account. In this case study, for example, there is a need for didactic training and coaching of medical specialists responsible for competence-oriented teaching and learning, which in turn must be used to enhance the competency of junior doctors undergoing training. Personnel and organisational development are thus interdependently linked.

#### 3.3. Evaluation

In view of the objectives of the Master's Degree Programme in Leadership in Medicine at the personnel and organisational development level, the set of evaluation tools is specially tailored to the degree programme and designed to facilitate action and reflection at different levels. The first level of course evaluation concerns participant satisfaction and the possible need for readjustment. It was based on a survey conducted using the ILIAS learning management system of the Bundeswehr University in Hamburg as well as on verbal evaluation rounds after every classroom-based phase. Academic project supervision during the project-based distance learning phase was also the subject of written and verbal evaluation. The second level of evaluation focuses on participant self-reflection, which is recorded for each module in the course portfolio, from motivation to self-assessment of personal competency development. The third level leads into institutional quality management as described under 3.2., where personnel and organisational development are combined. The evaluation of the projects carried out at the Federal Armed Forces Hospital Hamburg permits the assessment of the effectiveness and sustainability of academically verified innovation to be integrated in measures to develop the teaching quality of the Master's Degree Programme in Leadership in Medicine. The multitude of perspectives afforded by the set of evaluation tools, which surpass the standard course-related evaluation approaches, firstly reveal “noticeable” personal development among the participants and, secondly, highlight the potential of the Federal Armed Forces Hospital Hamburg as a “learning organisation”.

## 4. Discussion

The highlighted milestones in the process of professionalising medical specialty training confirm the nationwide development requirements and potential outlined in the position paper by the German Association for Medical Education and offer plausible solutions. After forming the basis by defining a competency profile with learning objectives, it must be communicated and evaluated. After all, quality assurance is necessary on an institutional and nationwide level [1]. Achieving this aim, however, will require a change in the culture and system in the institutions that provide follow-on training as well as in the people actively involved in medical follow-on training.

What sets apart the Master's degree programme from other degree programmes (MBA) with an often economic focus is the possibility of prioritisation in medical follow-on and advanced training on the basis of leadership fundamentals. The specific profile is based on medical specialty training being identified as a leadership task and thus the need to honour the didactic professionalism of (future) trainers. The related potential of a medical specialty training programme that is yet to be (further) developed was embraced by the version of the degree programme described. Particular importance is attached to the medical-didactic approach, role models and professional conduct as well as system-based teaching and learning [12]. The “project products” of the modules consist of the participants' learning outcomes and thus, from the institution's perspective, represent value added, which serves as proof of the effective interlinking of personnel and organisational development. 

The time commitment of 125 teaching units per module requires the support of the employer with regard to granting leave of absence for classroom-based phases and project implementation during the distance learning phases, which, however, the institution stands to benefit from. Cooperation across departments in changing project teams improves networking and collegial relationships in the hospital, which radiates to the organisation's entire leadership, training and change culture. The multitude of perspectives afforded by the set of evaluation tools, which surpass the standard course-related evaluation approaches, firstly reveal “noticeable” personal development among the participants and, secondly, highlight the potential of the Federal Armed Forces Hospital Hamburg as a “learning organisation”.

## 5. Conclusions

This type of project-based academic supervision described supports the follow-on training of doctors in two ways. Firstly, in addition to learning about leadership fundamentals and appropriate academic approaches, the participants undergo didactic professionalisation, which they, as multipliers, take with them to their organisations. Secondly, thanks to the many "project products" they create, the participants contribute to both an improved organisational framework and also to the process of appropriately adapting in-house follow-on training concepts to current educational requirements. At this point, personnel and organisational development become interdependently linked, thus enabling graduates of the Master's Degree Programme in Leadership in Medicine to rise to future challenges in everyday clinical practice. 

## Competing interests

The authors declare that they have no competing interests. 

## Figures and Tables

**Table 1:**
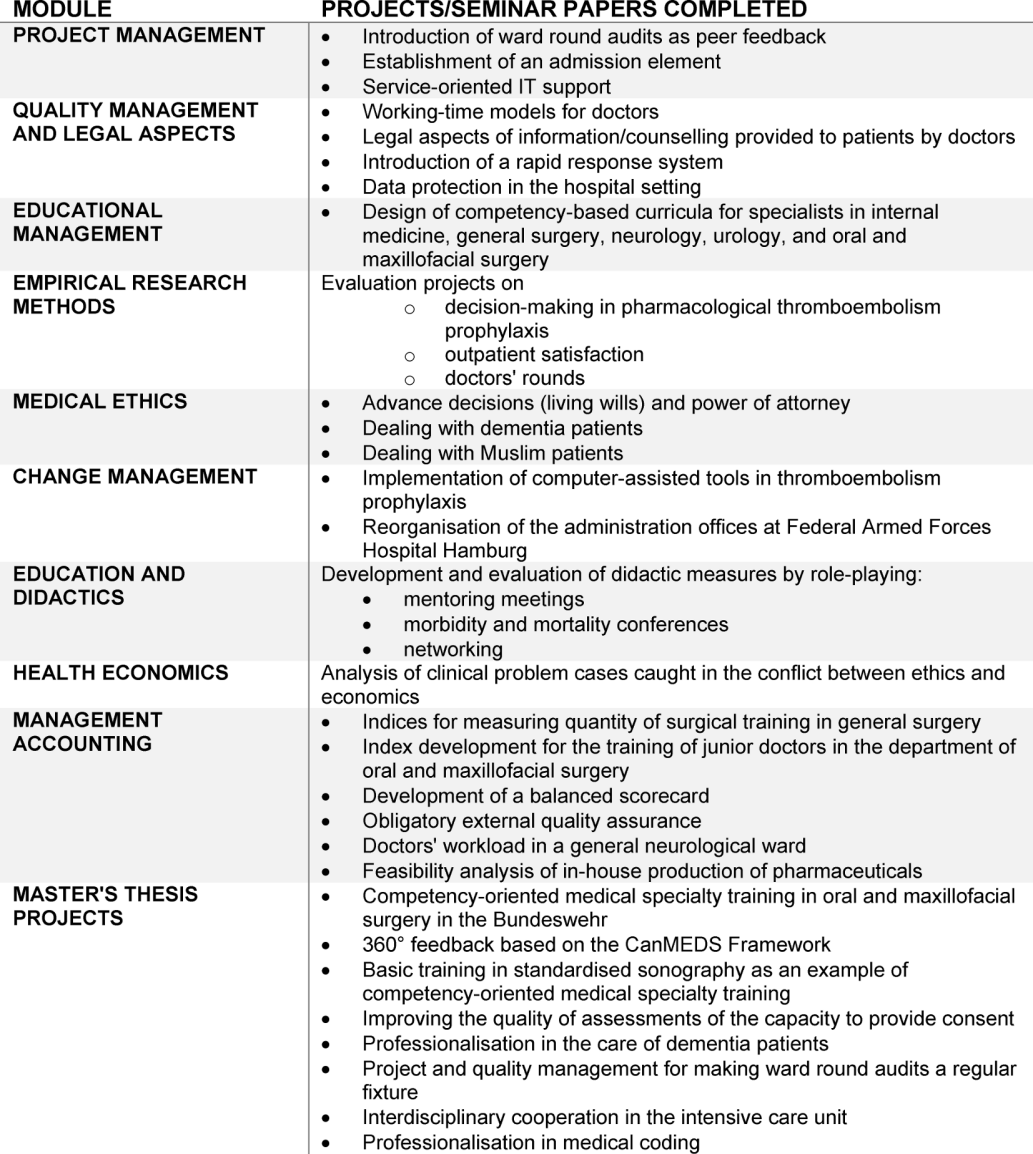
Selection of completed projects and seminar papers as well as planned Master's thesis projects as part of the modules
